# Pre-radiotherapy ctDNA liquid biopsy for risk stratification of oligometastatic non-small cell lung cancer

**DOI:** 10.21203/rs.3.rs-2688927/v1

**Published:** 2023-03-22

**Authors:** Nicholas P. Semenkovich, Pamela P. Samson, Shahed N. Badiyan, Gregory Vlacich, Hayley B. Stowe, Yun E. Wang, Rachel Star, Siddhartha Devarakonda, Ramaswamy Govindan, Saiama N. Waqar, Clifford G. Robinson, Bruna Pellini, Aadel A. Chaudhuri

**Affiliations:** 1 Division of Endocrinology, Metabolism, and Lipid Research, Department of Medicine, Washington University School of Medicine, St. Louis, Missouri; 2 Department of Radiation Oncology, Washington University School of Medicine, St. Louis, Missouri; 3 Siteman Cancer Center, Washington University School of Medicine, St. Louis, Missouri; 4 Tempus Labs Inc, Chicago, Illinois; 5 Division of Oncology, Department of Medicine, Washington University School of Medicine, St. Louis, Missouri; 6 Department of Thoracic Oncology, Moffitt Cancer Center and Research Institute, Tampa, FL, USA; 7 Department of Oncologic Sciences, Morsani College of Medicine, University of South Florida, Tampa, FL, USA.; 8 Division of Biology and Biomedical Sciences, Washington University School of Medicine, St. Louis, Missouri; 9 Department of Genetics, Washington University School of Medicine, St. Louis, Missouri; 10 Department of Biomedical Engineering, Washington University in St. Louis, St. Louis, Missouri; 11 Department of Computer Science and Engineering, Washington University in St. Louis, St. Louis, Missouri

## Abstract

The optimal treatment for patients with oligometastatic non-small cell lung cancer (NSCLC) remains unclear. Some patients with oligometastatic disease can experience prolonged remission after locally consolidative radiation therapy (RT), while others harbor micrometastatic disease (below current limits of detection by imaging) that may benefit from further prioritization of systemic therapy. To better risk-stratify this population and identify the patients most likely to benefit from locally consolidative radiation therapy, we performed a multi-institutional cohort study of patients with oligometastatic NSCLC undergoing liquid biopsy analysis of circulating tumor DNA (ctDNA).

Among this real-world cohort of 1,487 patients undergoing analysis (using the Tempus xF assay), a total of 1,880 ctDNA liquid biopsies along with paired clinical data were obtained across various timepoints. Approximately 20% (n=309) of patients had ctDNA obtained prior to RT and after their diagnosis of oligometastatic disease. Samples were de-identified and analyzed for mutational burden and variant frequencies of detectable deleterious (or likely deleterious) mutations in plasma.

Patients with undetectable ctDNA before RT had significantly improved progression-free survival and overall survival compared to patients with detectable ctDNA prior to RT. In patients that received RT, 598 pathogenic (or likely deleterious) variants were identified. ctDNA mutational burden pre-RT and ctDNA maximum variant allele frequency (VAF) pre-RT were both significantly inversely correlated with both progression-free (*P* = 0.0031 for mutational burden, P = 0.0084 for maximum VAF) and overall survival (*P* = 0.045 for mutational burden, P = 0.0073 for maximum VAF). Patients without detectable ctDNA prior to RT had significantly improved progression-free survival (*P* = 0.004) and overall survival (*P* = 0.03) compared to patients with detectable ctDNA prior to RT.

These data suggest that in patients with oligometastatic NSCLC, pre-radiotherapy ctDNA analysis can potentially identify the patients most likely to benefit from locally consolidative RT and experience prolonged progression-free and overall survival. Similarly, ctDNA may be useful to identify those patients with undiagnosed micrometastatic disease, in whom it may be appropriate to prioritize systemic therapy.

## Introduction

Oligometastatic non-small cell lung cancer (NSCLC) offers a unique opportunity for personalized liquid biopsy-guided therapies. While NSCLC patients with widespread metastatic disease are incurable and generally have poor outcomes, patients with a limited metastatic burden of disease can sometimes achieve prolonged remission after definitive management of the primary tumor and metastatic sites through a combination of systemic agents and local consolidative therapies such as RT [1–3].

However, identifying patients who have truly oligometastatic disease and are most likely to benefit from locally consolidative radiation therapy is challenging. Many patients with perceived oligometastatic NSCLC on imaging likely harbor undetected widespread metastatic disease (micrometastatic disease) below the limit of detection of current imaging technologies. Establishing a new liquid biopsy biomarker to segregate those patients with truly oligometastatic disease from those with widespread micrometastatic disease could alter treatment approaches. Patients with evidence of micrometastatic disease could be triaged to earlier systemic therapies or enrollment in clinical trials — in addition to sparing the costs, systemic therapy breaks, and potential side effects associated with local consolidative therapies. Similarly, clinicians could provide more concrete guidance to patients regarding the possibility of prolonged remission, and potentially offer more aggressive locally consolidative treatment in truly oligometastatic patients who do not harbor liquid biopsy evidence of micrometastatic disease.

We have previously shown that post-RT plasma circulating tumor DNA (ctDNA) is powerfully prognostic in localized NSCLC [4,5]. Here, we hypothesized that ctDNA analysis could be applied earlier (pre-RT) to risk-stratify patients with oligometastatic NSCLC and enable patient-personalized determination of local consolidative radiotherapy versus systemic therapy.

## Results

Median follow-up time after initial blood collection for liquid biopsy analysis was 10.3 months. Across all ctDNA assays, 3,503 pathogenic or likely pathogenic variants were identified (1.8 variants/sample, mean). Of the sub-cohort of 309 patients who underwent liquid biopsy after the diagnosis of oligometastatic disease and before RT, 48% (n=151) experienced progressive disease and 11% (n=34) died during the study period. ctDNA quantitation was based on the detection of pathogenic or likely pathogenic variants in plasma. ctDNA was detected in 74% of oligometastatic NSCLC patients prior to RT (n=230) while the remaining 26% (n=79) had no detectable ctDNA pre-RT. Among the 230 ctDNA-detectable patients, 76% (n=175) had 1–3 variants, while the remainder (n=55) had ≥4 pathogenic or likely pathogenic variants present.

**Figure 1:**
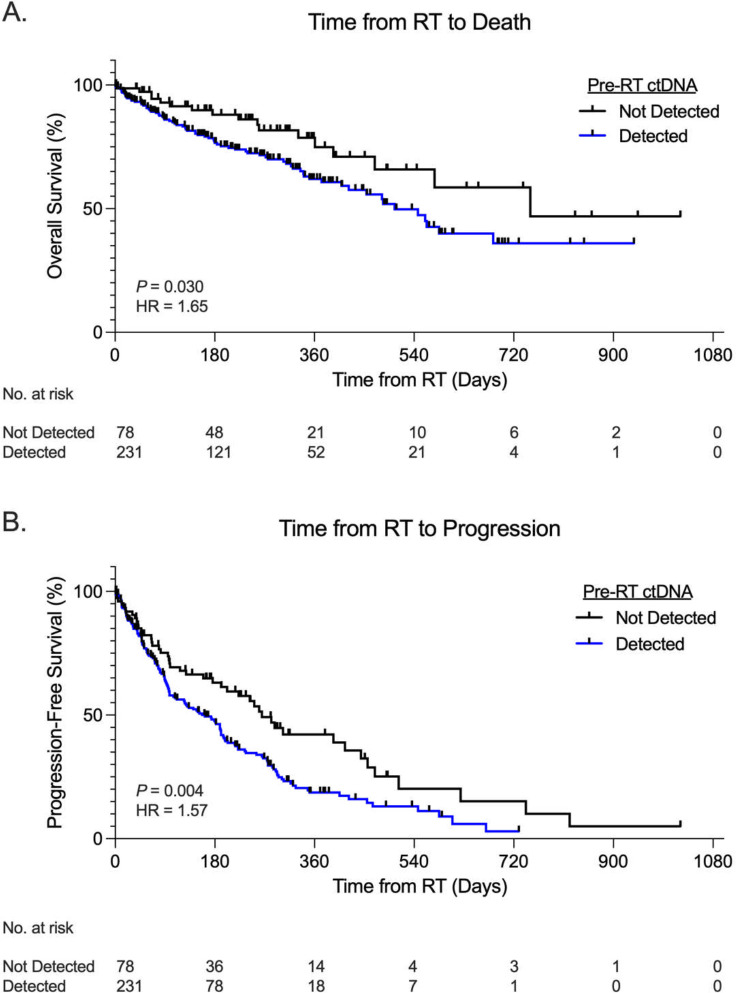
Survival stratified by pre-radiotherapy ctDNA detection in oligometastatic NSCLC Kaplan-Meier curves demonstrating both progression-free survival (A) and overall survival (B) in oligometastatic NSCLC patients stratified by ctDNA variant detection status prior to radiotherapy. P values were calculated by the log-rank test for trend and HRs by the Mantel-Haenszel method.

Overall survival was significantly worse in oligometastatic NSCLC patients with detectable ctDNA from pre-RT liquid biopsies, as compared to those without detectable ctDNA pre-RT, with a median OS of 16.8 months versus 25 months (p=0.030, hazard ratio [HR]=1.65, confidence interval [CI]=1.05–2.61) [Figure 1A]. Similar findings were also observed for PFS, which was worse in patents with detectable ctDNA pre-RT, with a median PFS of 5.4 months versus 8.8 months (p=0.004, HR=1.57, CI=1.15–2.13) [Figure 1B].

**Figure 2:**
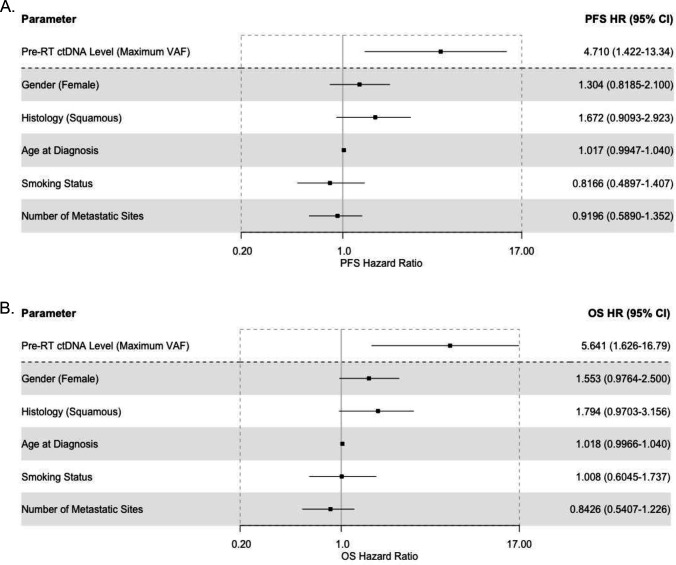
Multivariate Cox regression modeling of survival outcomes in oligometastatic NSCLC including the maximum ctDNA variant allele frequency Multivariate Cox regression modeling was performed for (A) progression-free survival and (B) overall survival with parameters including the maximum ctDNA variant allele frequency (VAF) prior to radiotherapy, as well as clinically relevant co-variates.

ctDNA levels (defined by the variant allele frequency) demonstrated significant risk correlations, with the maximum pre-RT ctDNA VAF associated with increased risk of both disease progression (p=0.0084) [Figure S1A] and death (p=0.0073) [Figure S1B]. These findings were corroborated by multivariate Cox proportional hazards modeling for PFS (p=0.04, PFS HR=4.71, CI=1.42–13.34) [Figure 2A] and OS (p=0.005, HR=5.64, CI=1.63–16.79) [Figure 2B]. Notably, multivariate modeling did not show significant impacts of other clinical parameters, including gender, age at diagnosis, smoking status, squamous histology, and number of metastatic sites. Additionally, the ctDNA mutational burden (the number of detectable pathogenic or likely pathogenic variants) was significantly associated with risk for both PFS (p=0.006, HR=1.17, CI=1.06–1.27) [Figure S2A] and OS (p=0.003, HR=1.16, CI=1.04–1.26) [Figure S2B].

## Discussion

The definition of the oligometastatic disease state has remained frustratingly subjective since its original proposal in 1995 by Hellman and Weichselbaum [11]. Locally focused treatment for disease control was initially only considered in select NSCLC patients with a solitary metastasis in either the brain or adrenal gland [12]. More recently, phase 2 studies in patients with up to 3 or 5 metastatic lesions have shown improved survival outcomes when treated with locally ablative radiotherapy [1–3]. Current trials are now testing whether this radiation-oriented paradigm may also benefit patients with even greater numbers of metastatic lesions [2,13], highlighting the need for more precise patient selection approaches. Our current work suggests that a pre-RT ctDNA liquid biopsy could serve as the first precision biomarker to objectively redefine oligometastatic disease, which would empower oncologists to provide more concrete advice to patients regarding RT for disease control and enable prioritization of systemic therapy for patients with ctDNA evidence of aggressive micrometastatic disease.

Indeed, to our knowledge, this study represents the largest ever real-world analysis of liquid biopsies in oligometastatic NSCLC, leveraging a multi-institutional dataset of 1,487 patients who underwent 1,880 liquid biopsies. Our analysis reveals that ctDNA testing performed pre-RT can risk-stratify those patients with truly oligometastatic NSCLC from those who likely harbor widespread micrometastatic disease (below current imaging limits of detection). Our modeling shows that the risk of disease progression and survival are informed by ctDNA quantitation, whether by mutational burden or the overall amount of ctDNA represented by VAF.

This approach should be prospectively evaluated in a clinical trial that redefines oligometastatic NSCLC to include a discrete liquid biopsy metric encompassing a low or undetectable ctDNA level. This technique may also be valuable for those patients who undergo curative-intent treatment for earlier stages of NSCLC, but subsequently develop oligometastatic disease (deemed “oligorecurrence”) and are weighing individualized treatment decisions.

### Limitations

This was a real-world study, with data collected from multiple clinical sites including both academic and community practices. Clinical data including metastases and RT were provided by the managing clinicians. Given incomplete data regarding the exact timing of oligometastatic disease diagnosis and RT, we explicitly focused on a sub-cohort where a liquid biopsy was definitively performed prior to RT administration in oligometastatic NSCLC patients. Progression status was determined by individual clinician assessments and did not follow a standard criterion. Although this introduces clinical heterogeneity into the dataset, it may also more accurately capture real-world clinical practice patterns and suggests broader extensibility of our clinical-correlative liquid biopsy findings. Moreover, our significant PFS data was corroborated by similarly significant OS data in this real-world cohort.

In conclusion, this study suggests that pre-RT ctDNA may be a powerful biomarker to accurately identify micrometastatic disease in patients with oligometastatic NSCLC. Earlier risk stratification using this liquid biopsy biomarker could support future clinical trials to enable personalized decision-making based on per-patient ctDNA risk profiles. Patients with high-risk ctDNA profiles could undergo systemic therapy prioritization and potentially escalation (avoiding systemic therapy breaks related to RT and potential RT toxicities), while patients with undetectable ctDNA or low ctDNA risk profiles could potentially be offered locally consolidative stereotactic radiotherapy.

## Methods

All analyses were performed using de-identified patient data. The study was exempt from institutional review board evaluation and informed consent given the de-identified nature of the data.

### Cohort Selection

We leveraged a multi-institutional real-world cohort consisting of 1,487 patients from both academic and community practices who were diagnosed with oligometastatic NSCLC. Peripheral blood samples were collected for liquid biopsy analysis between 2016 and 2022. The cohort mean age (SD) was 64.7 years (10.1), with similar numbers of male and female patients (784 female [53%], 703 male [47%]) [Table 1]. Approximately 73% of the patients had adenocarcinoma, 18% had squamous cell carcinoma, and 9% did not have histological subtyping available or had another subtype of NSCLC. Every patient underwent liquid biopsy and analysis using the Tempus xF assay at different timepoints, for a total of 1,880 ctDNA assays [Table 2]. All patients were reported by the treating physicians to have metastatic disease; we sub-selected a cohort for this analysis with metastases in 1–5 organ systems (n=309 patients), where a ctDNA liquid biopsy was definitively obtained after the diagnosis of oligometastatic disease, but prior to radiotherapy.

### Liquid Biopsy

The Tempus xF liquid biopsy assay is a laboratory developed test (LDT) designed to detect oncogenic and resistance mutations in cell-free DNA. The assay detects single nucleotide variants (SNVs), insertions/deletions (indels), rearrangements, copy number variations (CNVs), and microsatellite instability (MSI) with high sensitivity and specificity [6]. ctDNA results were analyzed for variants using VarDict [7] and characterized as pathogenic or likely pathogenic (P/LP) following ACMG/AMP guidelines for variant classification, and determined by SnpEff [8]. We excluded variants considered benign, likely benign, or having conflicting evidence. Variants were quantified per patient, along with the maximum variant allele frequency (VAF). Fusions and CNVs were identified using SpeedSeq [9] and CNVkit [10], respectively. Patient-level data of time to outcomes, ctDNA mutational burden, VAF, and other parameters used in this study are provided in Supplementary Table 1.

### Statistical Analysis

Median follow-up time was determined using the reverse Kaplan-Meier method. Outcomes for overall survival (OS) and progression-free survival (PFS) were both calculated from the start of RT (i.e., time from RT to death and time from RT to progression). Progression was reported by the managing clinicians. Kaplan-Meier curve p-values represent the log-rank test. Hazard ratios for OS and PFS in Kaplan-Meier analyses reflect the Mantel-Haenszel test. Multivariate Cox regression p-values were calculated using the Wald test. Data were managed using Apache Superset and statistical analyses were performed using GraphPad Prism version 9.5.0 and R version 4.2.2.

## Supplementary Material

Supplement 1

## Figures and Tables

**Table 1:** Cohort characteristics

Characteristic	Entire Cohort (n=1,487)	Sub-cohort (n=309)
Age, mean (SD), y	64.7 (10.1)	63.1 (10.1)
Sex		
Male	703 (47.3%)	149 (48.2%)
Female	784 (52.7%)	160 (51.8%)
Race and ethnicity		
Asian	93 (6.3%)	20 (6.5%)
Black	150 (10.1%)	38 (12.3%)
White	835 (56.1%)	176 (57.0%)
Other	44 (3.0%)	11 (3.5%)
Unknown	365 (24.5%)	64 (20.7%)
Primary Tumor Histology		
Adenocarcinoma	1,078 (72.5%)	233 (75.4%)
Squamous	267 (18.0%)	50 (16.2%)
Other/Not Specified	142 (9.5%)	26 (8.4%)
Metastatic sites, mean* (SD, range)	1.3 (0.64, 1–8)	1.3 (0.56, 1–5)
PD-L1 Status		
Positive	302 (20.3%)	59 (19.1%)
Negative	202 (13.6%)	48 (15.5%)
Unknown	983 (66.1%)	202 (65.4%)
Smoking Status		
Current/Former Smoker	1,006 (67.7%)	221 (71.5%)
Never Smoker	323 (21.7%)	73 (23.6%)
Unknown	158 (10.6%)	15 (4.9%)
Documented Radiotherapy	975 (66%)	309 (100%)

The sub-cohort was defined as those patients who underwent a liquid biopsy ctDNA test prior to receiving radiation therapy and after their diagnosis of oligometastatic NSCLC. Data are presented as the number (percentage) of patients, unless otherwise noted.

* Metastases were reported at the organ system level. All patients were reported by investigators to have metastatic disease, but specific counts of metastatic organ systems were unavailable in 293 (19.7%) of the entire cohort, excluding them from the sub-cohort.

**Table 2:** Genomic alterations detected in ctDNA

Characteristic	Entire Cohort	Sub-cohort
Liquid Biopsies Performed	1,880	434
Variants Detected, (mean per ctDNA test)	38,546 (20.5)	9,069 (20.9)
Pathogenic / Likely Pathogenic	3,503 (9.1%)	828 (9.1%)
Conflicting Evidence	17 (0.04%)	10 (0.11%)
Benign/Likely Benign	30,911 (80.2%)	7,254 (80.0%)
Uncertain Significance	4,115 (10.7%)	977 (10.8%)
Fusions Detected^†^	18	4
Copy Number Variations Detected^†^	34	11

All patients underwent at least one ctDNA liquid biopsy test.

† Fusion and CNV numbers reflect only pathogenic or likely pathogenic findings. For fusions, these encompassed *CD74-ROS1, EML4-ALK, FGFR2-TACC2, KIF5B-RET, SOD2-ROS1,,* and *SPTBN1-ALK*. For CNVs, these included *CCNE1, EGFR, ERBB2, MET,* and *MYC.*

## Data Availability

The data supporting this study’s findings are within the article and supplemental files. Supplementary Table 1 contains deidentified patient-level data (including time to outcomes, ctDNA mutational burden, and other parameters) that can be used to reproduce the findings of this study.
