# Supplementary material for: Pre-radiotherapy ctDNA liquid biopsy for risk stratification of oligometastatic non-small cell lung cancer
Source: Res Sq. 2023 Mar 22:rs.3.rs-2688927. Preprint. [Version 1] doi: 10.21203/rs.3.rs-2688927/v1 (PMC10055612; doi:10.21203/rs.3.rs-2688927/v1)
Supplement: Supplement 1 [file NIHPPrs2688927v1-supplement-1.pdf]

Supplementary Figure 1: Survival stratified by pre-radiotherapy ctDNA levels in oligometastatic NSCLC

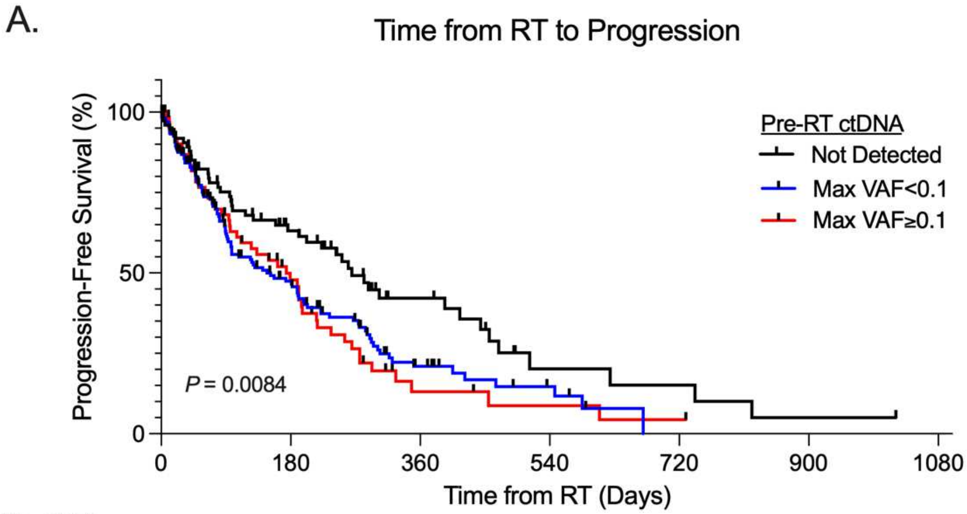

No. at risk

Not Detected	78	36	14	4	3	1	0
Max VAF <0.1	167	54	14	5	0	0	0
Max VAF ≥0.1	64	23	4	2	1	0	0

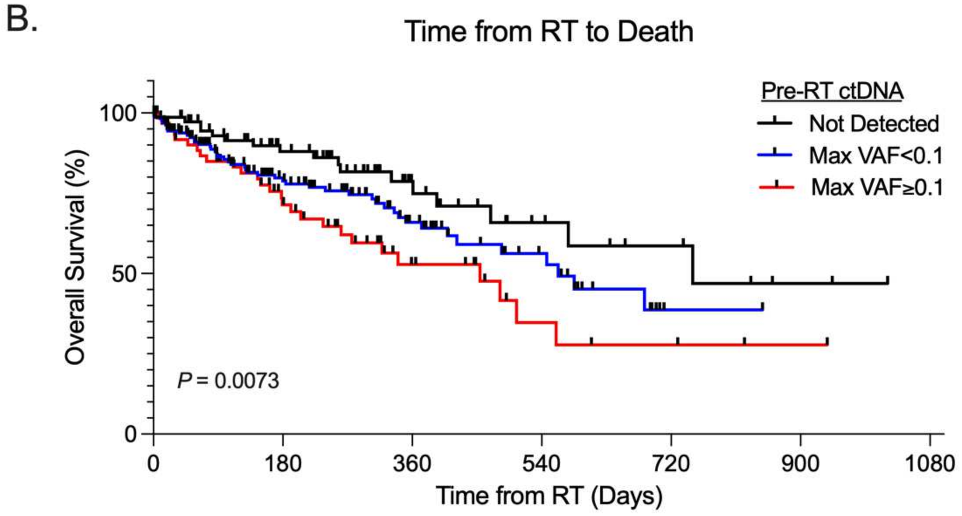

No. at risk

Not Detected	78	48	21	10	6	2	0
Max VAF <0.1	167	87	38	16	1	0	0
Max VAF ≥0.1	64	34	14	5	3	1	0

Kaplan-Meier curves demonstrating both progression-free survival (A) and overall survival (B) in oligometastatic NSCLC patients stratified by pre-radiotherapy ctDNA maximum variant allele frequency (VAF) levels. P values were calculated by the log-rank test for trend.

Supplementary Figure 2: Multivariate Cox regression modeling of survival outcomes in oligometastatic NSCLC including ctDNA mutational burden

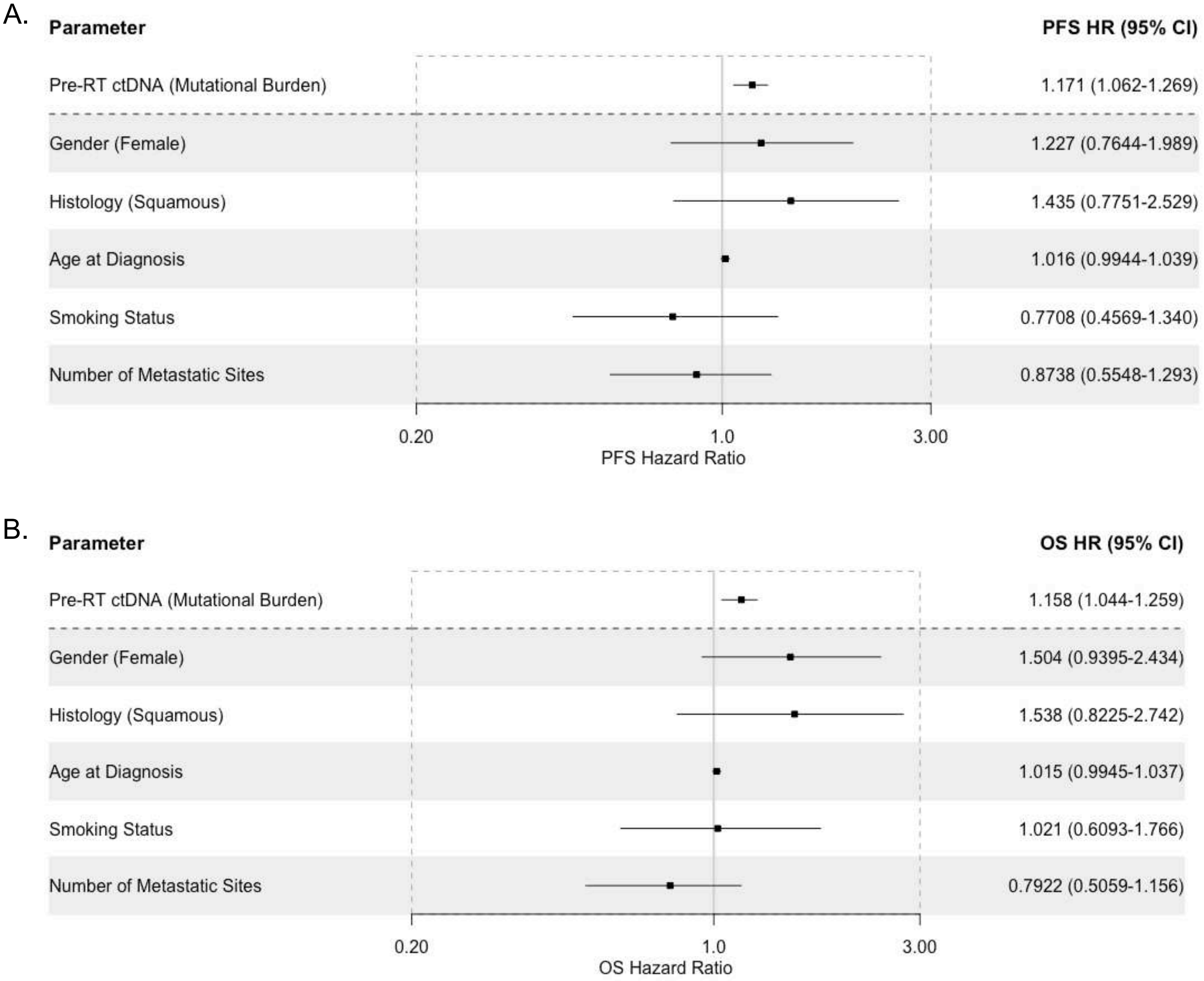

Multivariate Cox regression modeling was performed for (A) progression-free survival and (B) overall survival with parameters including the number of detected mutations in ctDNA (mutational burden) prior to radiotherapy, as well as clinically relevant co-variates.

Supplementary Table 1.

oligometastatic_NSCLC_summarized_dataset.csv: Anonymized data corresponding to the sub-cohort (n=309) necessary to reproduce all analyses in this work, including the ctDNA mutational burden, ctDNA variant allele frequencies, time to progression, time to death, and clinical and demographic parameters.
